# Proteomic profiling of human amnion for preterm birth biomarker discovery

**DOI:** 10.1038/s41598-021-02587-3

**Published:** 2021-11-30

**Authors:** Maurizio Bruschi, Martina Bartolucci, Andrea Petretto, Francesca Buffelli, Xhuliana Kajana, Alessandro Parodi, Riccardo Carbone, Ezio Fulcheri, Luca Antonio Ramenghi, Isabella Panfoli, Giovanni Candiano

**Affiliations:** 1 Laboratory of Molecular Nephrology, IRCCS Istituto Giannina Gaslini, Via Gerolamo Gaslini, 5, 16147 Genoa, Italy; 2 Core Facilities—Clinical Proteomics and Metabolomics, IRCCS Istituto Giannina Gaslini, Genoa, Italy; 3 Fetal-Perinatal Pathology Unit, IRCCS-Istituto Giannina Gaslini, Genoa, Italy; 4 Neonatal Intensive Care Unit, IRCCS-Istituto Giannina Gaslini, Genoa, Italy; 5 Department of Paediatric Science, University of Genoa, Genoa, Italy; 6 Dipartimento Di Farmacia (DIFAR), Università Di Genova, Genoa, Italy

**Keywords:** Biomarkers, Preterm birth, Mass spectrometry

## Abstract

Spontaneous preterm birth (PTB) complicates about 12% of pregnancies worldwide, remaining the main cause of neonatal morbidity and mortality. Spontaneous preterm birth PTBs is often caused by microbial-induced preterm labor, mediated by an inflammatory process threatening both maternal and newborn health. In search for novel predictive biomarkers of PTB and preterm prelabor rupture of the membranes (pPROM), and to improve understanding of infection related PTB, we performed an untargeted mass spectrometry discovery study on 51 bioptic mid zone amnion samples from premature babies. A total of 6352 proteins were identified. Bioinformatics analyses revealed a ranked core of 159 proteins maximizing the discrimination between the selected clinical stratification groups allowing to distinguish conditions of absent (FIR 0) from maximal Fetal Inflammatory Response (FIR 3) stratified in function of Maternal Inflammatory Response (MIR) grade. Matrix metallopeptidase-9 (MMP-9) was the top differentially expressed protein. Gene Ontology enrichment analysis of the core proteins showed significant changes in the biological pathways associated to inflammation and regulation of immune and infection response. Data suggest that the conditions determining PTB would be a transversal event, secondary to the maternal inflammatory response causing a breakdown in fetal-maternal tolerance, with fetal inflammation being more severe than maternal one. We also highlight matrix metallopeptidase-9 as a potential predictive biomarker of PTB that can be assayed in the maternal serum, for future investigation.

## Introduction

Preterm birth (PTB), defined by the World Health Organization (WHO) as delivery before 37 weeks of gestation, affects 5–18% of pregnancies^1^ and remains a major health and economics issue, due to neonatal mortality and morbidity. PTB is caused by diverse pathologic processes, among which decidual vascular disease or premature senescence; decline in progesterone action; uterine overdistension and microbial-induced inflammation^2^. The latter is the only one associated to spontaneous preterm delivery, in fact one in three preterm infants has a subclinical intra-amniotic infection^2^.

Sequelae of amniotic membrane infection include rupture of the amniotic membrane before the onset of labor, which can occur both at term (prelabor rupture of the membranes, PROM) and preterm (preterm pre-labor rupture of membranes, pPROM), in turn the most common cause of PTB as the consequence of excess inflammation and a breakdown in fetal-maternal tolerance^3,4^. pPROM complicates about 3% of pregnancies and causes one third of PTB, significantly contributing to perinatal morbidity and mortality^5–9^. About 30% of pPROM cases are secondary to microbial invasion of the amniotic cavity (MIAC), while 70% result from other causes^10–12^. In the former case, pPROM is mostly due to damage of chorioamniotic membranes by infiltrating inflammatory cells, secondary to MIAC. Pattern recognition receptors, such as toll-like receptors (TLRs) sense the presence of microbes inducing the inflammatory process mediated by chemokines, cytokines, and proteases^13^. Microbes may come from the lower genital tract or hematogenous dissemination, although the essential mechanisms have not yet been clarified^14^. Transplacental passage from periodontal disease has been hypothesized^15^. A common finding in pPROM is histological subclinical (HCA), or acute chorioamnionitis, an inflammation of the chorion, amnion and placenta. Intrauterine infection in fact triggers both maternal and fetal inflammatory responses, referred to as the fetal (FIR) and the maternal inflammatory response (MIR), respectively. In particular, MIR is the inflammatory response of the placenta^16^, HCA, that extends into the chorion, amnion or decidua. A persistent HCA, associated with MIAC, elicits subsequent FIR, clinically defined as a fetal cord blood plasma interleukin (IL)-6 concentration > 11 pg/mL. FIR is an inflammation infiltrating the chorionic plate, umbilical cord (funisitis) and fetal blood vessels (vasculitis of chorioamniotic vessels, vasculitis of umbilical vein and artery) and associated with poor neonatal outcomes including low birth weight, and sepsis^17–19^.

Risk scoring systems, attempting to stratify high or low risk of PTB according to maternal factors have been developed. Ultrasound measurement of cervical length at 20–24 weeks of gestation has been utilized^20^, however these methods have low-detection and high false-positive rates^21^.

In this preliminary study, by untargeted proteomics and bioinformatic analyses, we compared the protein expression of 51 human bioptic amnion samples from newborns in the ICU of Giannina Gaslini Pediatric Hospital, to identify the proteins that maximize the discrimination between the different clinical traits chosen. These were the FIR classes and histological characteristics (presence or absence of membrane phlogosis, funisitis, vasculitis of the umbilical vein or artery or vasculitis of chorioamniotic vessels), in function of MIR classes.

## Results

**Figure 1:**
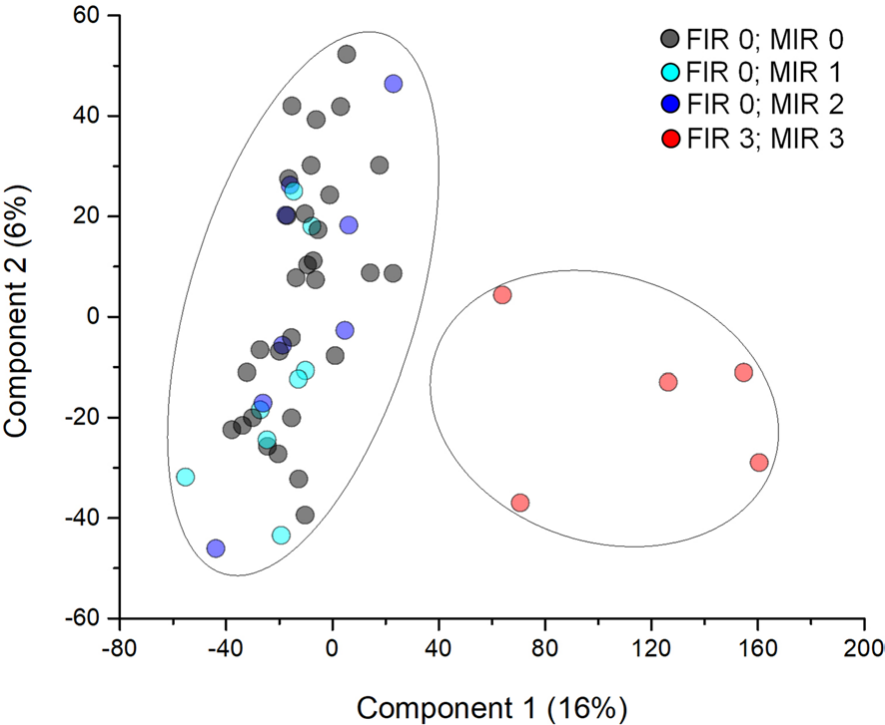
Multidimensional scaling (MDS) of bioptic amnion proteome profiles. Scatter plot of MDS analysis of FIR 3-MIR 3 (red circles), FIR 0-MIR 2 (blue circles), FIR 0-MIR 1 (cyan circles) and FIR 0-MIR 0 (black circles) samples. Ellipsis indicates 95% confidence interval. Plot shows clustering of two distinct groups (FIR 3 and FIR 0 samples).

The proteome profile of the 51 amnion samples collected from mid zone was determined by Orbitrap mass spectrometry. A total of 6352 proteins were identified, 4633 (72.9%) of which were overlapping between FIR 0 and FIR 3 samples. Notably, 1565 proteins (24.6%) or 154 (2.4%) were exclusive for FIR 0 or FIR 3, respectively (Supplemental Fig. 1). Notwithstanding the high protein identity overlap between the two groups, multidimensional scaling analysis evidenced a clear discrimination between FIR 0 and FIR 3 samples (Fig. 1). The weighted gene co-expression network analysis (WGCNA) clusters in a module the proteins sharing the same co-expression profile, that are deemed to be in a functional relationship with each other^22^. We used the WGCNA to identify those modules/protein expression profiles functionally associated to the selected clinical traits. WGCNA revealed a total of 5 modules encompassing proteins with similar co-expression profiles (Supplemental Fig. 2). An arbitrary color was chosen for each module, to distinguish them. The number of proteins included in each module were 255 (red), 388 (blue), 1366 (cyan), 1402 (magenta) and 3029 (green), respectively. The blue module showed closer relationships with FIR 0, MIR 0, grade 0 membrane phlogosis (PM), and with absence of funisitis, vasculitis of chorioamniotic vessels, vasculitis of umbilical vein and artery type samples, respectively with r = 0.8, 0.48, 0.48, 0.84, 0.8, 0.93 and 0.79 (correlation among MIR 0 and PM grade 0 (PM 0) was not statistically significant). By contrast, the red module showed closer relationship with FIR 3, MIR 3, grade 3 PM (PM 3), presence of funisitis and chorioamniotic vessels, umbilical vein and artery vasculitis, with r = 0.94, 0.85, 0.85, 0.84, 0.8, 0.93 and 0.79, respectively (all correlations were statistically significant). No other statistically significant correlations were present between modules and clinical traits. The Spearman’s coefficient profile between all proteins and all clinical traits, after Z-score normalization, was depicted by a heatmap diagram (Supplemental Fig. 3). Visual inspection of WGCNA and its associated cluster analysis demonstrates the ability of the proteomic profile of the proteins associated with the red/blue modules to distinguish between the presence or absence of funisitis, umbilical vein or artery vasculitis and vasculitis of chorioamniotic vessels, i.e.: between FIR 0 or MIR 0 or MP 0 and FIR 3 or MIR 3 or MP 3 samples.

**Figure 2:**
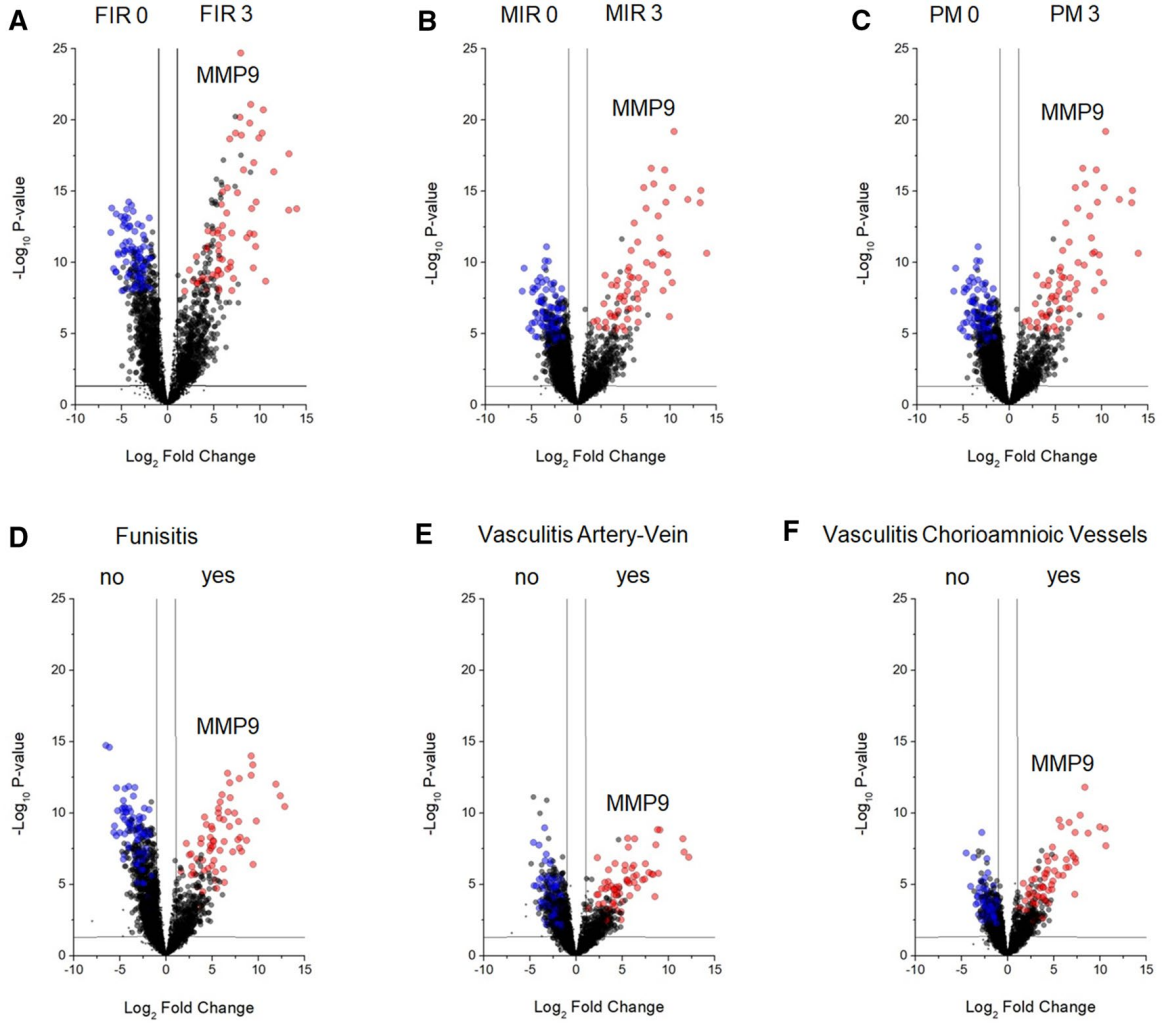
Volcano plots of all comparison. Volcano plot for (**A**) FIR 0 vs FIR 3; (**B**) MIR 0 vs MIR 3; (**C**) PM 0 vs PM 3; (**D**) absence vs presence of funisitis; (**E**) absence vs presence of umbilical artery or vein vasculitis; (**F**) absence vs presence of vasculitis of chorioamniotic vessels. Black, blue and red circles indicate the changes for the non-significant or significant of previously described groups, respectively. Black line indicates the limits of statistically significant. Black circles above the black line indicate the proteins with an identity < 70%.

**Figure 3:**
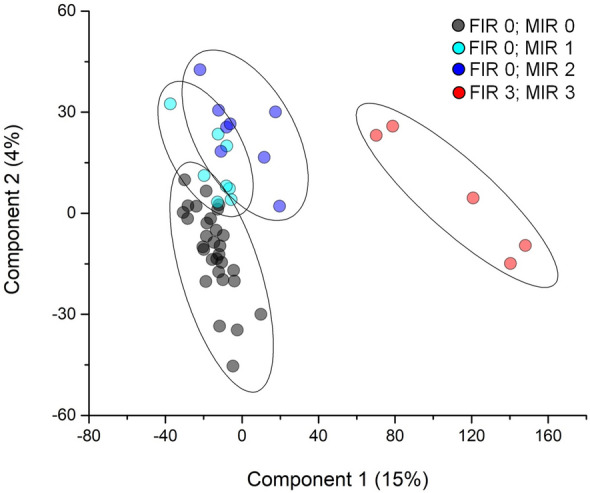
Partial least square discriminant analysis (PLS-DA) of proteins core list. Scatter plot of PLS-DA analysis of FIR 3-MIR 3 (red circles), FIR 0-MIR 2 (blue circles), FIR 0/MIR 1 (cyan circles) and FIR 0/MIR 0 (black circles) samples. Ellipsis indicates 95% confidence interval. Plot shows clustering of two distinct groups (FIR 3 and FIR 0 samples). These proteins can clearly discriminate between the FIR 0 and FIR 3 samples. Besides, a good discrimination between FIR 0-MIR 0 and other FIR 0-MIR 1–2 groups are present.

We applied the T*-*test to identify the proteins that best distinguished the above comparison. This analysis revealed 1585, 1071, 1071, 1256, 1309 and 534 discriminative proteins, respectively, in the comparison of FIR 0 vs FIR 3, MIR 0 vs MIR 3, PM 0 vs PM 3 with the presence or absence of funisitis, vasculitis of umbilical vein and/or artery and vasculitis of chorioamniotic vessels (Fig. 2 and Supplemental Fig. 4). 455 proteins overlapped between all the comparisons^23^ (Supplemental Fig. 4). Besides, the two-way ANOVA test was applied to identify the proteins that best distinguished between FIR 0 and FIR 3 samples, stratified in function of MIR grade. Such analysis revealed 1299 discriminative proteins. Finally, the combined use of the T-test, two-way ANOVA, PLS-DA and SVM revealed 159 proteins maximizing the discrimination between the different clinical stratification groups of amnion samples (Supplemental Table 1). In particular, 65 or 94 proteins were enriched in samples with FIR 3 or FIR 0, respectively (Supplemental Fig. 5). Moreover, the same core list of proteins also maximized the discrimination between FIR 0 and FIR 3, stratified in function of MIR value (Fig. 3). Notably, while all the FIR 3 samples came from subjects presenting rupture of membranes, the other FIR 0 samples presenting rupture of membranes (pPROM) did not distinguish from those with intact membranes, therefore did not determine a distinct cluster (Figs. 1 and 3). In all statistical comparisons (ANOVA and T-test) MMP9 was the protein displaying the highest P-value. Moreover, in SVM/PLS-DA MMP9 was the protein occupying the first rank position/displaying the highest VIP score, respectively (see Supplemental Table 2 for details). For this reason, MMP9 resulted the most promising biomarker for the discrimination between FIR 0 and FIR 3 stratified in function of MIR value and of presence or absence of funisitis, vasculitis of umbilical vein or artery and vasculitis of chorioamniotic vessels.

The wide diversity in structure and expression profiles among FIR 0 and FIR 3 samples, and their correlation with either MIR 0, PM 0 and absence of funisitis, vasculitis of umbilical vein or artery and vasculitis of chorioamniotic vessels or with MIR 3, MF 3 and presence of funisitis, vasculitis of umbilical vein or artery and vasculitis of chorioamniotic vessels, respectively, could imply their different biological role. To assess this, we performed Gene Ontology (GO) enrichment analysis based on cellular component, biological processes, molecular function, KEGG, Reactome and keywords annotation. GO analysis identified 396 significant enriched gene signatures. Among these, 295 and 101 were enriched in FIR 0 or FIR 3, respectively (Supplemental Table 2). In particular, 35 gene signatures were significantly changed above 95% of CI. These latter signatures were all enriched in FIR 3 samples. The GO enrichment analysis was visualized using a scatter plot (Supplemental Fig. 6). In the plot, the points located on the straight line passing through the coordinates (1_x_,1_y_) and (− 1_x_, − 1_y_) are the equally enriched signatures, while the distance from this line is proportional to the increase of signature enrichment. In particular, the points above or under the straight line are the GO annotations/pathways positively enriched in FIR 3 or in FIR 0, respectively. This GO enrichment analysis shows the significant changes of the various biological pathways associated to inflammation and regulation of immune and infection response. The results are summarized in a network diagram of biochemical pathways that were enriched in FIR 3 samples (Supplemental Fig. 7). In this network, circles and lines represent the biochemical pathways and their inter-connections, respectively. In addition, the different pathways were clustered in groups in function of their GO annotation (black ellipses). For example, the functional pathways activated include defense response to microorganisms, cytokine production and regulation, cellular assembly and organization, leukocyte activation and migration, phagocytosis, immune system processes, activation of matrix metalloproteases and regulation of proteolysis and extracellular matrix (ECM) degradation. Furthermore, the analysis identified the pathways facilitating molecular exocytosis and exosomal proteins. The GO enrichment analysis was also conducted for the core list of 159 proteins highlighted by the bioinformatic analysis (Supplemental Table 4). The results of this analysis are summarized in a network diagram showing a significant change in the same biological processes involving infection and inflammation, with neutrophil, metalloproteinase, endopeptidase, kinase and phosphorylation proteins activation and the perturbation of IL-17 and the chemokine signaling pathway as those highlighted by the analysis conducted on the total dataset (Supplemental Fig. 8).

**Figure 4:**
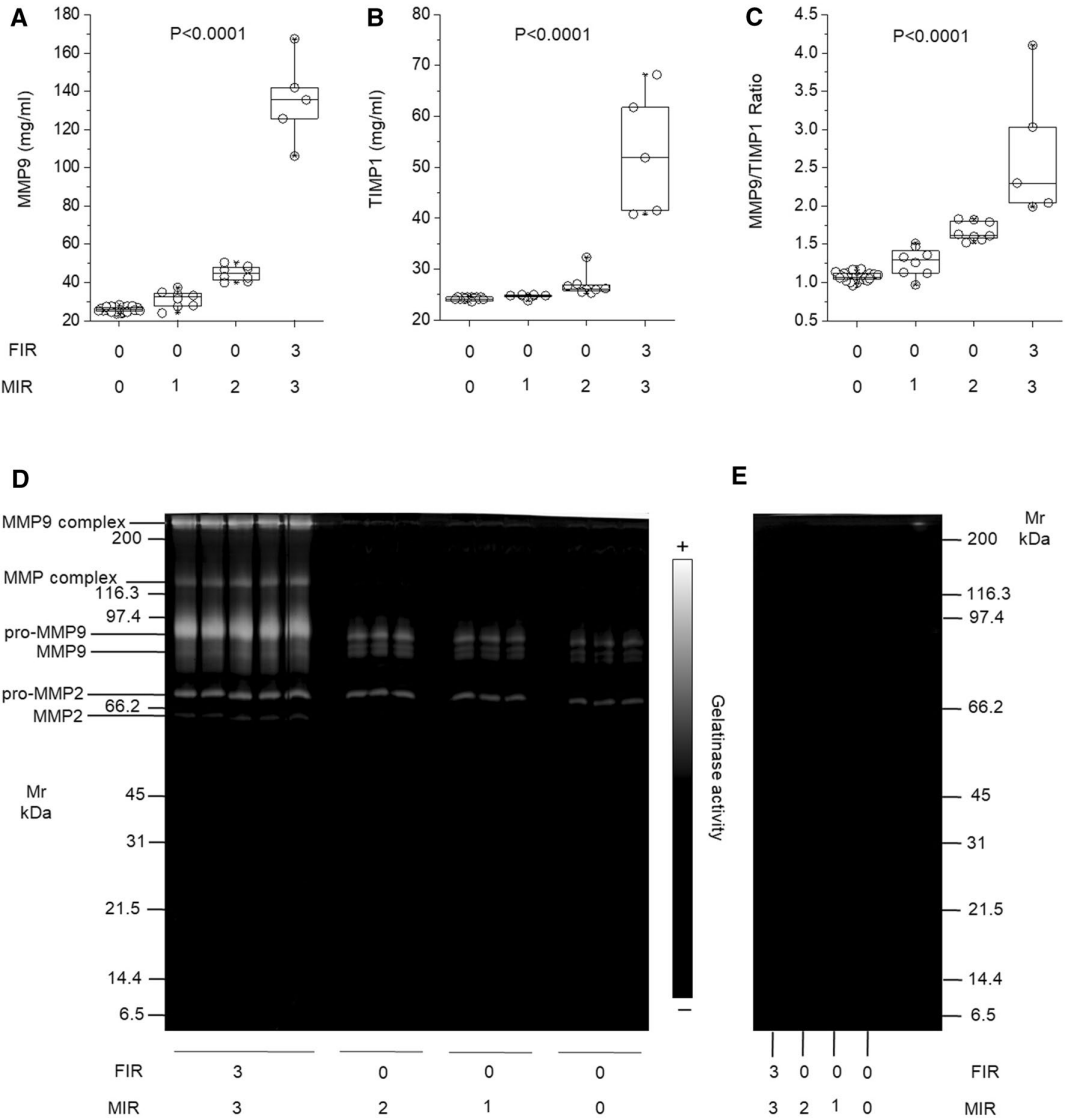
MMP9 and TIMP1 ELISA assay and zymogram of solubilized amnion bioptic samples. Box plots show the median and interquartile range value for (**A**) MMP9 and (**B**) TIMP1 proteins and (**C**) their ratio in all samples. MMP9 and TIMP1 were statistically more abundant in FIR 3-MIR 3 samples in comparison to all other samples (P < 0.0001). (**D**) full-length Zymogram gel shows the gelatinase activity of all FIR 3-MIR 3 and a randomized selection of the other solubilized bioptic amnion samples. (**E**) Full-length Zymogram of a randomized selection of one for each of the solubilized bioptic amnion samples incubated, during the digestion, overnight with 10 mM of EDTA. The enzymatic activity of all samples was completely inhibited. The ELISA assay and their enzymatic activity are in agreement with the data obtained in the proteomic analysis.

We used homemade ELISA assay to validate the change in levels of MMP9 and TIMP1 proteins in all amnion samples. Both proteins were statistically more abundant (P < 0.0001 for both ELISA assay) in FIR 3 compared to other groups (Fig. 4A,B). The median/IQR ng/ml values for MMP9 were 26.05 (25.3–26.9) (FIR 0-MIR 0), 32.35 (27.8–34) (FIR 0-MIR 1), 44.85 (41.92–47.6) (FIR 0-MIR 2) and 135.5 (125.7–141.9) (FIR 3-MIR 3). Moreover, the median/IQR ng/ml values for TIMP1 (inhibitor of MMP9) were 24.1 (23.8–24.5) (FIR 0-MIR 0), 24.8 (24.72–24.85) (FIR 0-MIR 1), 26.1 (25.87–26.8) (FIR 0-MIR 2) and 51.9 (41.5–61.8) (FIR 3-MIR 3). ROC curve analysis revealed an area under the curve (AUC) and confidence interval (CI) of 0.95 (0.9–1) and 0.95 (0.9–1) in the comparison of FIR 0 vs FIR 3 respectively for MMP9 and TIMP1. Besides, in both assays, considering the likelihood ratio, the association between high level of MMP9 or TIMP1 in amnion samples and FIR 3 resulted with a Likelihood ratio > 10. In addition, amnion samples with FIR 3-MIR 3 displayed higher MMP 9/TIMP-1 ratios compared with other groups (P < 0.0001; Fig. 4C).

Consistently with the results from the proteomic analysis and ELISA assay for MMP9 expression profile, we found a gelatinase activity statistically significantly more abundant in FIR 3/MIR 3 compared to other groups (P < 0.0001). Zymogram of amnion samples showed all forms of MMPs as gelatinase activity, as usually found with this technique^24–26^, thereby including the homodimer of the pro-MMP9 form (225 kDa), the pro-MMP9 complexed with neutrophil gelatinase-associated lipocalin (NGAL) form (130 kDa), the pro-MMP9 form (92 kDa), the active MMP9 (84 kDa), the pro-MMP-2 (72 kDa) and the active MMP2 (62KDa) (Fig. 4D). Notably, gelatinase activity was completely quenched by adding 10 mM EDTA (Fig. 4E), confirming that the visualized bands correspond to MMP activity.

## Discussion

Here we studied the protein expression profile of amnion proteins from a population of premature human newborns, stratified according to different histopathological patterns (FIR, MIR, funisitis, vasculitis of the umbilical vein or artery, or of the chorioamniotic vessels). Diagnostic criteria for the staging of the maternal and fetal inflammatory responses in intrauterine infections was done according to the consensus statement for placental lesions, established by the Amsterdam Placental Workshop Group^27^.

This preliminary study focused on the two extremes of fetal inflammation: FIR 0 and FIR 3, i.e. either absence or high fetal inflammation (necrotizing funisitis) respectively, in function of MIR. The study aimed at paving the way to subsequent studies investigating the two intermediate conditions, FIR 1 (chorionic vasculitis or umbilical phlebitis) and FIR 2 (involvement of the umbilical vein and umbilical arteries) that were underrepresented in our study population, therefore not statistically significant. It has been proposed that the amnion is the dominant structural component of the fetal amnio-chorial membrane and is primarily responsible of its integrity^28^.

The protein expression profiles of FIR 0 and FIR 3 clustered separately. Despite the high overlap in the protein identity, MDS analysis allowed a complete distinction of the two clinical groups. This implies that, although almost half of the identified proteins were identical, the expression pattern of many of them changed. Out of the 1299 discriminative proteins identified, the SVM analysis identified 459 core proteins, 159 of which maximized the discrimination between the different clinical stratification groups of amnion samples. The WGCNA identified the 5 co-expression modules that correlated or not with the histological data. A set of proteins in the red and blue modules were highly correlated with FIR 0 or FIR 3, respectively, by contrast there was no significant correlation with FIR 1 and 2 conditions.

FIR stage/severity is linked to progression of inflammation from the umbilical vein to arteries and to Wharton’s jelly, manifesting as funisitis (inflammation of the umbilical cord). Occurrence of FIR in our population, ranging between 24 and 33 weeks gestational age (GA), is consistent with the fetus ability to respond to bacterial colonisation, that starts from 21 weeks GA^20^.

Several proteomic analyses have been done on pPROM, that have identified proteins related to inflammation and infection. A study of the protein complement of human placental amniochorionic membrane reported the involvement of inflammation, infection, proteolysis and metabolic alterations, with membrane ECM degradation in both pPROM and PROM^29^. A Tandem Mass Tag-based approach to the proteome of the amniotic fluid from amniocentesis of women with late pPROM (between 34 and 37 weeks of gestation) found 138 proteins significantly changed, associated with acute inflammatory response in samples from women with MIAC and HCA^30^. In particular, the upregulated proteins included S100 proteins, histones, secreted neutrophil proteins as well as proteins associated to redox processes, while the downregulated proteins encompassed regulators of inflammatory response, such as thrombospondin-1^30^. Similar result underlying infection-associated HCA and MIAC in pPROM were obtained from another quantitative shotgun proteomic study on amniotic fluid from amniocentesis^31^. pPROM human placental tissues were studied by two-dimensional gel electrophoresis coupled with mass spectrometry and bioinformatics analysis, and it was found that the proteins with altered expression belonged to cytoskeleton, metabolism, and oxidative stress^32^. An increased concentration of neutrophil elastase was reported in the pPROM amniotic fluid^33^.

Interestingly, our data are consistent with the histology of placenta. In particular, PTB is frequently (~ 70%) associated with HCA, that is observed also in 50% of PROM. HCA is more common than clinical chorioamnionitis, which is characterised by maternal clinical symptoms^3,20^. Intrauterine infection can occur through haematogenous spread, but more commonly microbes ascend from the dysbiotic microbiome of the lower genital tract, when the relative abundance of protective Lactobacillus spp. is impaired^20^. The existence and role of a placental microbiota in spontaneous PTB is however still controversial^34^. The pathogen-associated molecular patterns activate the expression of toll-like receptors (TLR). Increased expression of TLR-2 and TLR-4 on chorioamniotic membranes increases the expression of proinflammatory cytokines and chemokines in the placenta, causing HCA^20^.

Notably, in the PLS-DA two component analysis, the samples in the FIR 0/MIR 2 group tended to move slightly from the more clustered samples related to the conditions FIR 0/MIR 0 and FIR 0/MIR 1 towards the clearly separated FIR 3 (necrotizing funisitis)/MIR 3 (necrotizing chorioamnionitis) cluster, confirming that the maternal inflammation is decisive in determining the outcome, i.e. PTB. Along this view, our data are consistent with the notion that FIR is more severe than MIR and more frequently associated with poor neonatal outcome^20^. The conditions that determine PTB would be a transversal event of maternal origin causing a breakdown in fetal-maternal tolerance, mostly secondary to maternal inflammatory responses. The latter are a reversal of the T-helper (Th)2 anti-inflammatory healthy placental microenvironment to Th1 microenvironment, associated with expression of proinflammatory cytokines (IFN-γ, IL-2 and IL-12) that stimulate labour, and spontaneous PTB with or without pPROM^3^. In fact, the placenta normally induces regulatory T cells (T_regs_) with immunosuppressive properties and M2-phenotype anti-inflammatory decidual macrophages^20,35^. The connection between maternal and fetal inflammation was also reported by a prospective cohort study of 25 women with pPROM^36^. When placental inflammation on the fetal side was present, both maternal blood and fetal umbilical cord blood IL-6 and IL-8 and fetal IL-1β, and TNF-α were elevated at delivery. FIR was also associated worse neurological outcome^36^. Bacterial colonisation of the placenta reverses this equilibrium, by stimulating the fetal hypothalamic-pituitary axis, with increase in fetal cortisol and Prostaglandins (PG) synthesis, in turn inducing placental CRH production^37^. The involvement of inflammatory pathways^31^ as well as inflammation-triggered proteolysis is well known in PTB, which strengthens the results of the present blind bioinformatic analysis. Both CRH and PG stimulate infiltration of inflammatory and matrix metallopeptidases (MMP) production, eliciting FIR^38^. Consistently, MMP9 was the protein in the first position of the core ranked list of the 159 proteins maximizing the difference among the clinical stratification groups. From an evolutionary point of view, the function of placental inflammatory response and ultimately PTB in case of MIAC would bear a survival value, as it allows the mother to reject the infected fetal allograft and maintain reproductive ability^39^. In turn, inflammation of the fetus may have a survival value as it contributes to mount the infant defence response against infection^34,40^*.* In fact, in our samples PTB showed no correlation with birth weight, degree of prematurity, eclampsia, gestosis, or intrauterine growth restriction (IUGR)^41^. Nor was there any correlation in our new-borns with any of the complications of prematurity, i.e. Necrotizing Enterocolitis (NEC), sepsis, cerebral/cerebellar hemorrhage, white matter punctate lesions.

The GO annotations enriched in amnions from fetuses with FIR 0/MIR 0 respect to FIR 3/MIR 3 were related to biological processes involved in defense response against microorganisms, cytokine production and regulation, cel assembly and organization, leukocyte activation and migration, phagocytosis, immune system processes, activation of matrix metalloproteases-regulation of ECM degradation. Moreover, the GO annotations enriched in the core list of highlighted proteins were related to infection and inflammation, neutrophil activation, MMPs, endopeptidase, kinases, protein phosphorylation, activation of IL-17 and chemokine signaling pathway. MMP9 was the protein best characterizing the distinction between FIR 0 and FIR 3 and MIR 0 and MIR 3. ELISA and zymogram analyses confirmed an increase MMP-9 activity. MMP9 proteolyzes the ECM and plays a role in leukocyte migration, cleaving type IV and V collagen and degrading fibronectin. MMP9 is expressed in amnion, chorion, decidua parietalis, and placental syncytiotrophoblasts^42^. The involvement of MMP-9 activation was suggested in some PROM cases^43^. Higher concentrations of MMP-9 were found in plasma of pPROM fetuses, respect to those born with intact membranes^44^. In particular, median MMP-9 concentrations in amniotic fluid form women with MIAC were higher than in those without microbial invasion, and among these, women with pPROM had higher MMP-9 concentrations than those with PROM with intact membranes^45,46^.

TIMP1 is a MMP inhibitor that acts by binding to its target, irreversibly inactivating the catalytic zinc cofactor. To validate the mass spectrometry and ELISA results, we measured the amnion concentrations of MMP9 and TIMP-1 by SDS-PAGE zymography, a highly sensitive technique able to detect both the zymogen and active form of MMP-9 and MMP -2, based on their gelatinase activity. We also evaluated the MMP-9/TIMP-1 ratios, to gather information regarding the net MMP activities. The increase in MMP-9 levels, MMP-9/TIMP-1 ratios and gelatinase activity in amnion FIR 3-MIR 3 samples, compared with other groups, would indicate that the inflammation was not balanced in the former. Increased MMP-9 expression would be a major contributor to amnion ECM degradation, triggering PTB. In fact, a change in either TIMP or MMP levels can cause a net change in specific MMP activity. MMP/TIMP imbalance has been implied in various pathological conditions^47^.

An imbalanced ratio between MMP-9:TIMP-1 mRNA in the progression and malignancy of canine Mast Cell Tumours was reported^48^.

Taken together, our findings are consistent with the hypothesis that MMP-9 is a potential biomarker of PTB for pPROM (correlated to FIR3/MIR3 in our samples) risk, as previously proposed^49^ Monitoring MMP9 in the maternal blood, as a biomarker, may allow the early identification of the risk of PTB in selected individuals. In fact, the present data were remarkably correlated with the placental histology routinely done in the clinical setting after PTB. On the other hand, histological examination can only be done a posteriori and moreover is strongly operator-dependent.

## Materials and methods

### Materials

All chemical compounds were of the highest chemical grade.

### Samples

Randomized selection of bioptic amnion samples from consecutive premature newborns (ranged between 24–34 weeks) in the ICU of Giannina Gaslini Pediatric Hospital were divided in four groups in function of their Fetal and Maternal Inflammatory response (FIR and MIR) grade.

The placentas were blindly read by two different pathologists:

1. 30 samples with FIR 0 and MIR 0.
2. 8 samples with FIR 0 and MIR 1.
3. 8 samples with FIR 0 and MIR 2.
4. 5 samples with FIR 3 and MIR 3.

FIR and MIR grades were established by histological examination conducted following the guide line of the Amsterdam Placental Workshop Group^27^ by two independent operators.

Moreover, 18, 1, 1, 5 amnion samples coming respectively from FIR 0/MIR 0, FIR 0/MIR 1, FIR 0/MIR 2 and FIR 3/MIR 3 subjects presented rupture of membranes. All Demographic and clinical characteristics are reported in Table 1. All newborns were born by cesarean delivery.

**Table 1:** Clinical data. Continuous data are reported as median and interquartile range.

	FIR 0-MIR 0	FIR 0-MIR 1	FIR 0-MIR 2	FIR 3-MIR 3
Sample size (51)	30	8	8	5
Gestational age week	31 (28–32)	30 (27–31)	28 (26–31)	28 (25–29)
Weight at birth gr	1390 (1030–1700)	1402.5 (995–1775)	1135 (840–1590)	1030 (830–1150)
APGAR 1’ score	6 (5–7)	6 (5–7)	6 (5–6)	5 (4–6)
APGAR 5’ score	8 (8–9)	8 (8–9)	8 (7–8)	8 (6–8)
Placental weight gr	367.5 (260–475)	275 (203.5–390)	257 (245–366)	198.5 (182–290.5)
Preterm birth with rupture of membranes yes/no	18/12	1/7	1/7	5/0
Complete steroid prophylaxis yes/no	25/5	7/1	2/2	4/1
Membrane phlogosis grade 0/1/2/3	30/0/0/0	0/8/0/0	0/0/8/0	0/0/0/5
Funisitis yes/no	0/30	0/8	1/7	4/1
Vasculitis of umbilical artery yes/no	0/30	0/8	1/7	3/2
Vasculitis of umbilical vein yes/no	0/30	0/8	1/7	4/1
Vasculitis of chorioamniotic vessels yes/no	0/30	0/8	2/6	4/1

The study was carried out in accordance with Italian and international ethical guidelines and were approved by the Liguria Regional Ethics Committee (number 033REG2015). Written informed consent was obtained from all subjects.

### Sample preparations

Human amnion membrane bioptic samples (about 2 × 2 cm of size) were taken from the mid zone of fresh placental tissue (not formalin fixed)^50^ and then homogenized on ice in 400 µl of solubilisation buffer (50 mm Tris–HCl pH 6.8, 2% w/v sodium dodecyl sulphate (SDS) supplemented with protease and phosphatase inhibitor cocktail) using IKA T10 Basic homogeniser (IKA Labortechnik, Staufen, Germany). The process required six cycles of homogenization of 20 s. Then the homogenized samples were centrifuged at 20,000×*g* for 60 min at 4 °C. The supernatant was removed and used for the study. Samples aliquots for mass spectrometry, ELISA and zymogram analysis were kept at − 80 °C until use.

### Protein identification by mass spectrometry (MS) analysis

#### Sample preparation

Solubilised, reduced and alkylated amnion samples (100 µg aliquots) were prepared for mass spectrometry analysis by FASP method^51^ using 30 k Vivacom filtration devices (Sartorius). Protein digestion was performed by adding trypsin and LysC (at a 1:50 and 1:100 ratio of enzyme to sample protein respectively, both in micrograms), mixing and incubating at 37 °C overnight. Protease activity was quenched by adding trifluoroacetic acid (TFA) at a final concentration of 1% and the resulting peptide mixture was clarified on StageTip with two C18 solid phase extraction disks (Empore) prepared by washing with 80% ACN with 0.1% TFA and 0.1% TFA in water, followed by loading of the acidified peptide mixtures. Peptides were eluted using 80% Acetonitrile with 0.1% TFA. The eluate was completely dried using a SpeedVac centrifuge at 30 °C and resuspended in 2% ACN and 0.1% FA.

Purified peptides were analyzed by a nano-UHPLC-MS/MS system using an Ultimate 3000 RSLC coupled to an Orbitrap Fusion Tribrid mass spectrometer (Thermo Scientific Instrument). Elution was performed with an EASY spray column (75 μm × 50 cm, 2 μm particle size, Thermo Scientific) at a flow rate of 250 nl/min with a 135 min non-linear gradient consisting of 3 min wash with 2% buffer B (80% ACN, 20% H2O, 5% DMSO and 0.1% FA), then increasing to 30% B over 97 min, with a further increase to 50% B in 20 min, followed by a 5 min wash at 80% B and a 10 min re-equilibration at 2% B.MS scans were acquired at a resolution of 120,000 between 375 and 1,500 m/z and an AGC target of 4.0E5.MS/MS spectra were acquired in the Orbitrap at a resolution of 30,000 and an AGC target of 5.0E5. Quadrupole isolation with a 1.8 m/z isolation window was used, and dynamic exclusion was enabled for 25 s.HCD was performed using 28% normalized collision energy. One microscan was used for both MS1 and MS2 events. Raw data were processed with MaxQuant software^52^ version 1.6.14.0. A false discovery rate (FDR) of 0.01 was set for the identification of proteins, peptides and PSM (peptide-spectrum match). For peptide identification a minimum length of 6 amino acids was required. Andromeda engine, incorporated into MaxQuant software, was used to search MS/MS spectra against Uniprot human database (release UP000005640_9606 March 2020). In the processing the Acetyl (Protein N-Term), Oxidation (M) and Deamidation (NQ) were selected as variable modifications and the fixed modification was Carbamidomethyl (C). Whole Mass spectrometry data are friendly available at ProteomeXchange Consortium via the PRIDE^53^ partner repository with the dataset identifier PXD028343 (Reviewer account details: Username: reviewer_pxd028343@ebi.ac.uk, Password: hdkPcOfk).

### MMP9 and TIMP1 ELISA

For MMP9 and TIMP1 ELISA assay, 100 µg aliquots of amnion samples were filtered with 500 Da Amicon Ultra centrifugal filter device in phosphate buffer saline (PBS). In homemade ELISA, 100 µl per well of 5 µg/ml of anti-human MMP9 (Rabbit polyclonal, SIGMA-Aldrich) or anti-human TIMP1 (Rabbit polyclonal, SIGMA-Aldrich) in phosphate buffer saline (PBS) were coated onto 96-well maxisorp nunc-immuno plates (ThermoFisher Scientific, MA, USA) overnight at 4 °C and blocking in 3% w/v BSA in PBS. After blocking, 100 μl of ultra-filtered samples per well were incubated overnight at 4 °C, washed three times with PBS and 0.05% v/v of tween-20 (PBS-T) and incubated 4 h with anti-human MMP9 (clone 2H4, SIGA-Aldrich) or anti-human TIMP1 antibody (clone 4D12, SIGMA-Aldrich). Then, plates were washed three times with PBS-T and incubated for 1 h with HRP-conjugated Mouse anti-Human IgG. At the end of incubation, samples were washed again three times with PBS-T. Finally, the peroxidase substrate (TMB, Bio-Rad) was added, and absorbance at 450 nm was measured using a xMark microplate Absorbance Spectrophotometer (Bio-Rad).

### Zymogram

Samples (10 µg proteins) were separated onto polyacrylamide gradient (8–16%) denaturing gels (SDS-PAGE) containing type A gelatine from Porcine skin (SIGMA-Aldrich). Following electrophoresis, the gel was rinse in Tris-buffered saline (TBS) and 2% (v/v) Triton-X100 and incubated with TBS and 5 mM CaCl_2_ overnight at 37 °C^24^. Enzymatic activity was visualized as a clear band against a blue background after staining with blue silver coomassie^24,54^. Notably, to quench the enzymatic activity the gel was incubated in TBS and 10 mM EDTA overnight at 37 °C^24^. Gel images were acquired and analysed by the ChemiDoc Touch Imaging System and Image Lab software respectively (Bio-Rad, Hercules, CA, USA).

### Statistical and bioinformatic analyses

The sample size was determined based on biological variability of samples in order to allow the identification of statistical change bearing a probability p = 0.05 and power = 80%^55,56^ in two-way ANOVA test with samples stratified in function of FIR and MIR grade ant T-test between zero and maximal grade of FIR, MIR, PM and absence and presence of funisitis, umbilical vein or artery vasculitis and vasculitis of chorioamniotic vessels. Each Label-free quantification data was log2 converted, and normalized^57^. After normalization and missing value replacing with normal distribution (width 0.3 and down shift 1.8), unsupervised hierarchical clustering (Multidimensional scaling) with *k*-means were used to identify outlier and samples similarity. Normalized dataset was used to construct the co-expression network using the weighted gene co-expression network analysis (WGCNA)^22^. Once chosen the appropriate β power parameter (with the value of independence scale set to 0.8), proteins were clustered into modules of co-expression profiles (50 proteins at least). Then, Spearman’s correlation and unsigned network type were used to identify the relationship between each module or intensity value of identified proteins and discrete (FIR, MIR, funisitis, phlogosis of membrane, umbilical vein or artery vasculitis, vasculitis of chorioamniotic vessels) or continuous (gestational age at birth and weight at birth) clinical traits indicators. Heatmap was used to show this result. In the heatmap each row represents a module or protein, and each column corresponds to a trait indicator. A pseudocolor scale depicts Spearman’s coefficient of each module/trait indicator, with red indicating a perfect positive correlation (+ 1), white no correlation (0) and blue perfect negative correlation (− 1). The tree dendrogram displays the results of an unsupervised hierarchical clustering analysis placing similar Spearman’s correlation coefficients values next to each other. Differentially expressed proteins between FIR 0 and FR 3 type samples were detected using a T-test. P-values for each protein were adjusted using the method of Benjamini–Hochberg^56^. Proteins were considered significantly differentially expressed when displaying an adjusted P-value ≤ 0.05, identified in at least 70% of one of groups and a fold time change ≥ 2. Volcano plot was used to quickly visualize the statistical differences and the cutoff lines were established using the function y = c/(x—× 0).

The same statistical significance cutoff was used in the comparison among the different grade of MIR and phlogosis of membrane, presence or absence of funisitis, vasculitis of the umbilical vein or artery or vasculitis of chorioamniotic vessels. By contrast, two-way ANOVA method was used to identify the statistically significantly changed proteins between FIR 0 and FIR 3 samples, stratified in function of MIR classes. To identify the proteins that maximize the discrimination between the above clinical traits, we combined the results obtained by T-test, two-way ANOVA, Partial Least Squire Discriminant Analysis (PLS-DA) and Support Vector Machine (SVM) learning^58^. In particular, for the latter analysis we utilized ANOVA method to optimize the feature selection and the four-fold cross-validation approach to estimate the prediction and classification accuracy. Besides, the whole matrix was randomly divided into two parts. One for learning (65%) and the other one (35%) to test the accuracy of prediction. The training was repeated until all possible combination of the subjects in the two groups are done (Origin Lab software). Gene Set Enrichment Analysis (GSEA)^59^ was done using whole dataset to identify a raked list of Gene Ontology (GO) annotation enrichment in FIR 0 or 3 samples . The rank was confined between − 1 and 1 corresponding to minimal and maximal enrichment in the two groups. The result of this analysis is shown by scatter plot and report the enriched GO annotation statically significant (P < 0.05 after multiple comparison correction). In the graph, the points located on the straight line passing through the coordinates (1_x_,1_y_) and (− 1_x_, − 1_y_) are the equally enriched signatures, while the distance from this line is proportional to the increase of signature enrichment in one of the two groups. In particular, the points above or under the straight line are the GO annotations/pathways positively enriched in FIR 3 or FIR 0, respectively. The resulting enriched rank proteins were uploaded to Cytoscape software using the EnrichmentMap^60^, ClusterMaker2^61^ and AutoAnnotate apps to construct and visualizing a protein–protein interaction network. Gene Ontology (GO) annotations were extracted from the Gene Ontology Consortium (http://www.geneontology.org/). Fisher’s GSEA^59^ was done for all modules in statistical correlation with at least one clinical trait and for the core panel of identified proteins. The proteome profile of the core panel were visualized using the heatmap diagram. In the heatmap each row represents a protein and each column corresponds to a sample. Normalized Z-scores of protein abundance are depicted by a pseudocolor scale with red indicating positive expression, white equal expression and blue negative expression, compared to each protein value. The tree dendrogram displays the results of unsupervised hierarchical clustering analysis, placing similar sample/proteome profile values next to each other. In the ELISA data analysis, the Kruskal–Wallis test was used to assess differences in the MMP9 and TIMP1 proteins levels among the FIR 0 and FIR 3 samples, stratified in function of MIR grade. Results were expressed as medians and interquartile range (IQr). A value of P < 0.05 after Dunns correction was considered statistically significant. Receiver operating characteristic (ROC) curves were generated to assess the diagnostic efficiency of both assays for the discrimination of FIR 0 and FIR 3 samples. Youden's index and Likelihood ratio^62^ were used, respectively to identify the cutoff and the diagnostic performance of the tests. Statistical analyses were performed using the last version of software package R available at the time of the experiments and OriginLab software.

## Supplementary Information

Supplementary Information 1.

Supplementary Information 2.
