# Supplementary material for: Proteomic profiling of human amnion for preterm birth biomarker discovery
Source: Sci Rep. 2021 Nov 30;11:23144. doi: 10.1038/s41598-021-02587-3 (PMC8633292; doi:10.1038/s41598-021-02587-3)
Supplement: Supplementary file 1 — Supplementary Information 1. [file 41598_2021_2587_MOESM1_ESM.pdf]

SUPPLEMENTAL FIGURES

PROTEOMIC PROFILING OF HUMAN AMNION FOR PRETERM BIRTH BIOMARKER DISCOVERY

Maurizio Bruschi[1*], Martina Bartolucci[2*], Andrea Petretto[2], Francesca Buffelli[3], Xhuliana Kajana[1], Alessandro Parodi[4], Riccardo Carbone[5], Ezio Fulcheri[3], Luca Antonio Ramenghi[4], Isabella Panfoli[6#] and Giovanni Candiano[1#].

[1]Laboratory of Molecular Nephrology, IRCCS Istituto Giannina Gaslini, Genoa, Italy

[2]Core Facilities – Clinical Proteomics and Metabolomics, IRCCS Istituto Giannina Gaslini, Genoa, Italy

[3]Fetal-Perinatal Pathology Unit, IRCCS-Istituto Giannina Gaslini, Genoa, Italy

[4]Neonatal Intensive Care Unit, IRCCS-Istituto Giannina Gaslini, Genoa, Italy

[5]Department of Paediatric Science, University of Genoa, Italy

[6]Dipartimento di Farmacia (DIFAR), Università di Genova, Genoa, Italy

*These two authors have equally contributed as first author

#These two authors have equally contributed as last authors

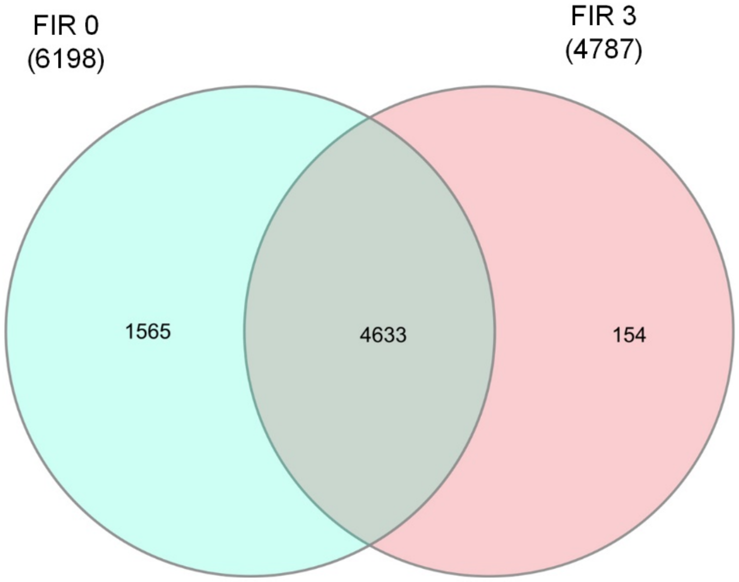

Supplemental Figure 1. Venn diagram of the identified proteins. Venn diagram show common and exclusive proteins. Numbers represent distinct proteins.

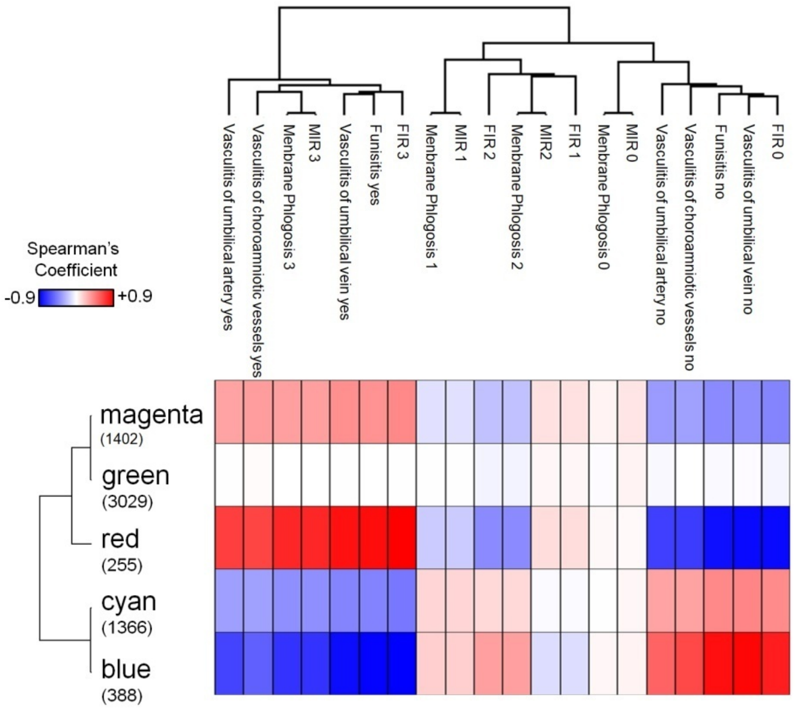

Supplemental Figure 2. Module identification and their relationship with clinical traits. Heatmap shows the module-trait weighted relationship between the identified modules and the indicator trait of samples. The color scale shows module-trait Spearman's correlation from – 0.9 (blue) to 0.9 (red). Each row represents a module and each column a clinical trait. The tree dendrogram (top and left) displays the results of an unsupervised hierarchical clustering analysis placing similar correlation coefficient values next to each other. Number of proteins in each module is shown in brackets

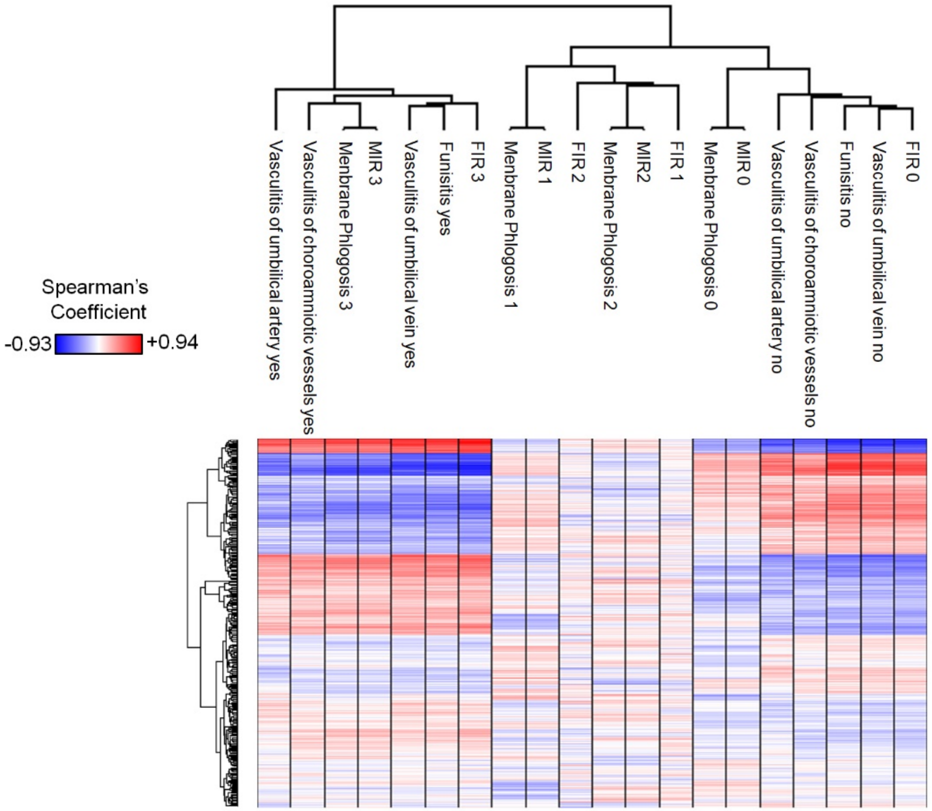

Supplemental Figure 3. Heatmap of Spearman's Correlogram of all proteins identified by mass spectrometry. In the diagram the Pearson's coefficients were converted in a pseudocolor scale ranging from -0.93 (blue) to 0.94 (red). Each row represents a protein and each column a clinical trait. The tree dendrograms (top and left) display the results of an unsupervised hierarchical clustering analysis placing similar correlation coefficient values next to each other.

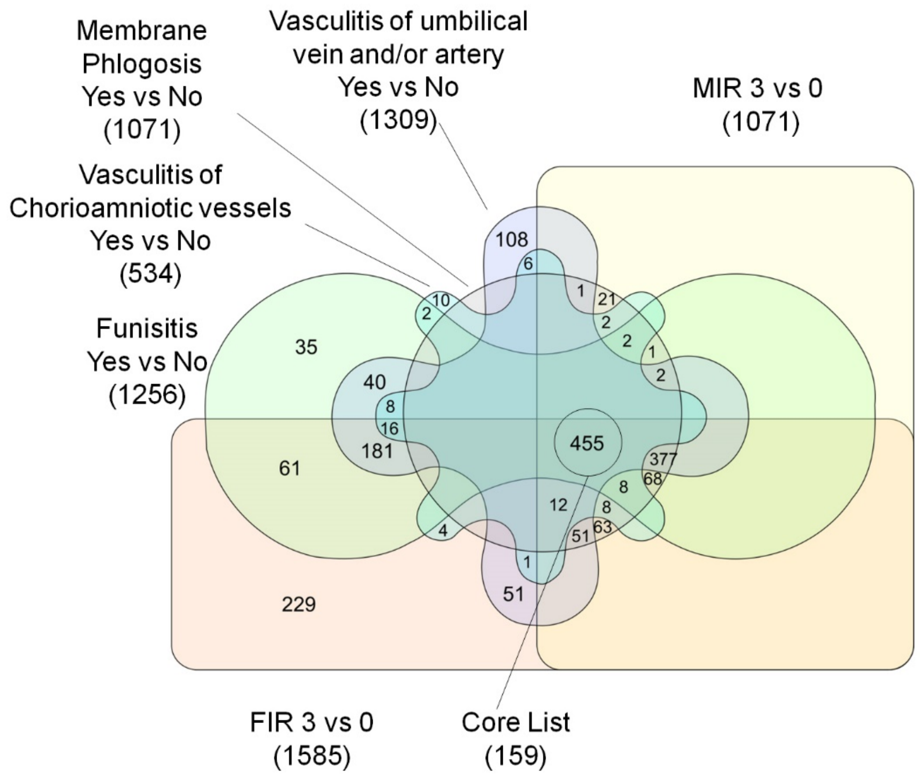

Supplemental Figure 4. Venn diagram of univariate statistical analysis results. Venn diagram shows common and non-common proteins resulting from T-tests for all comparisons performed in this study. Numbers represent the distinct proteins in the respective overlapping and not-overlapping areas.

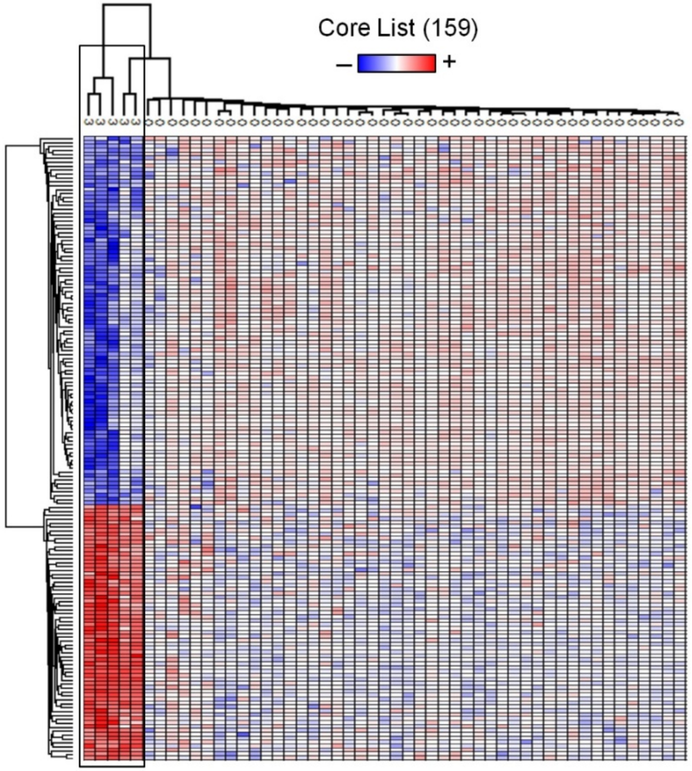

Supplemental Figure 5. Heatmap of the 159 proteins core list. Heatmap of the proteome profile of 159 proteins highlighted by the statistical analysis. Each row represents a protein and each column a sample. Normalized Z-scores of protein abundance are depicted using a pseudocolor scale (red, white and blue indicating positive equal and negative expression, respectively) compared to each protein value. The dendrogram displays unsupervised hierarchical clustering analysis. The similar sample/proteome-profile values are next to each other. These proteins can clearly discriminate between the FIR 0 and FIR 3 samples.

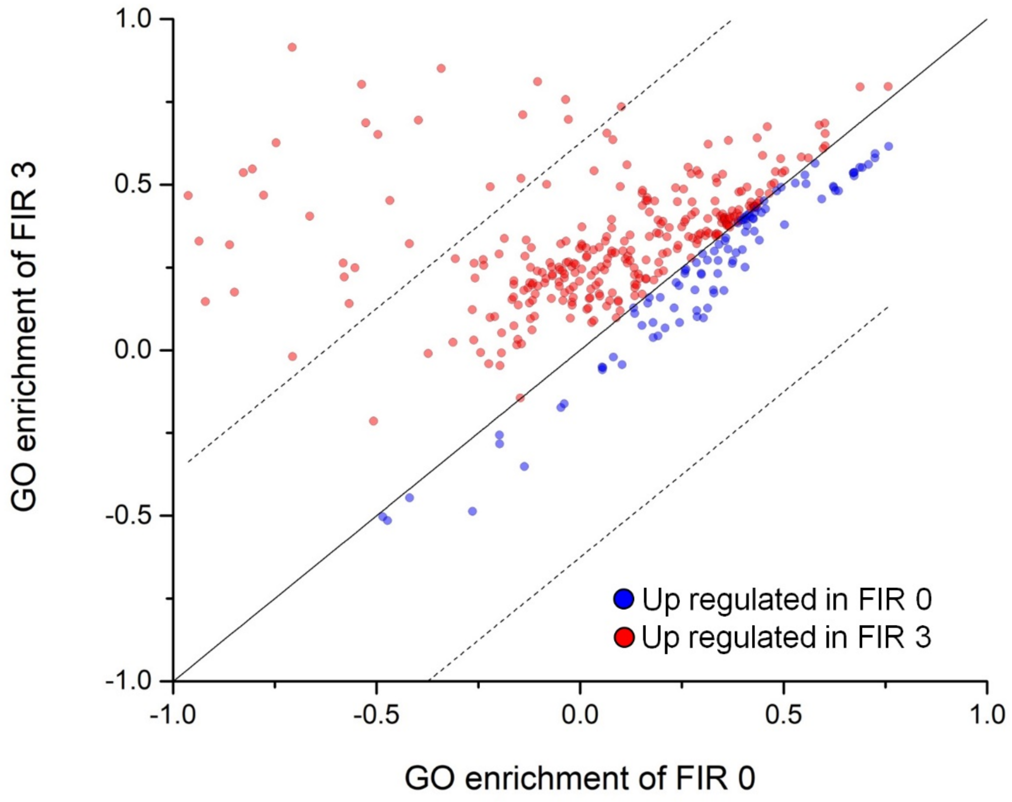

Supplemental Figure 6. Two-dimensional Gene ontology annotation enrichment analysis. Plot shows the enriched signatures in FIR 3 and FIR 0 samples. In the graph, the points located on the straight line drawn through the coordinates (1x,1y) and (-1x,-1y) represent the equally enriched signatures, while those above or under this line are positively enriched in FIR 3 or FIR 0, respectively. Besides, the points located between the two dotted line correspond to the signature displaying an enrichment score within the 95% of confidence interval (see detail in Supplemental table 2).

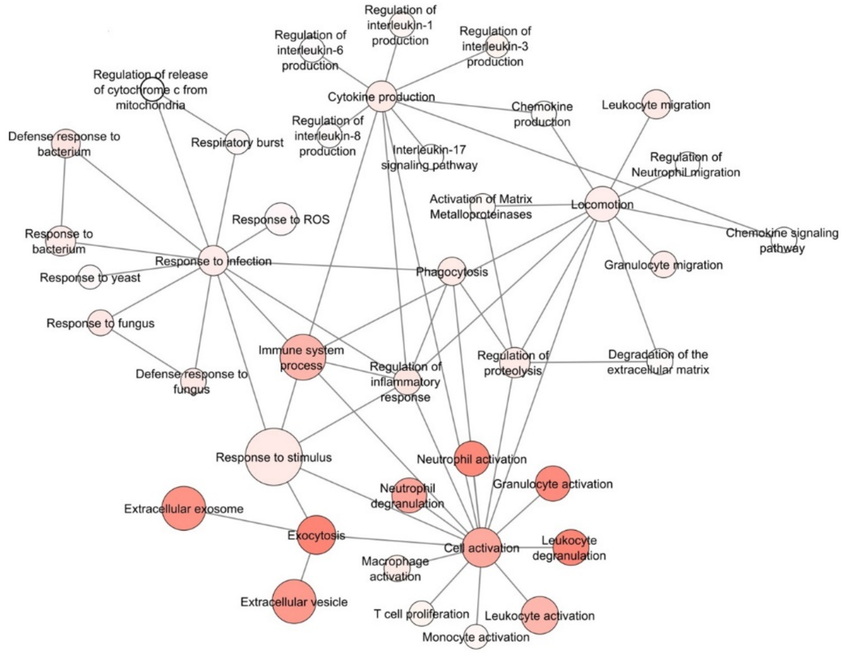

Supplemental Figure 7. Network of gene ontology enrichment results. The diagram shows the zoom-in of the enrichment results, mapped as a network. Nodes and edges represent, respectively, the biological processes and their interaction. The color intensity of each protein (node) indicates the grade of enrichment in FIR 3 versus FIR 0 samples while the node size is representative of their p-value.

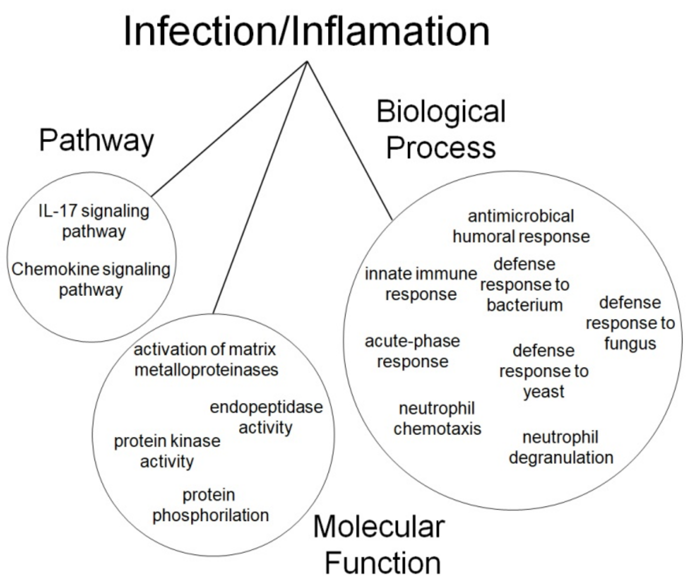

Supplemental Figure 8. Network diagram of gene ontology (GO) enrichment analysis of core list of highlighted proteins. The diagram summarizes the enrichment results of the list of 159 core proteins highlighted by bioinformatics analysis, mapped as a network. The statistically significant GO annotation changes were clusterized according to their GO type (see Supplemental Table 4 for detail).
